# Supplementary material for: Synthetic Cyclic C5-Curcuminoids Increase Antioxidant Defense and Reduce Inflammation in 6-OHDA-Induced Retinoic Acid-Differentiated SH-SY5Y Cells
Source: Antioxidants (Basel). 2025 Aug 28;14(9):1057. doi: 10.3390/antiox14091057 (PMC12466566; doi:10.3390/antiox14091057)
Supplement: Supplementary file 1 [file antioxidants-14-01057-s001.zip › Figure S3.pdf]

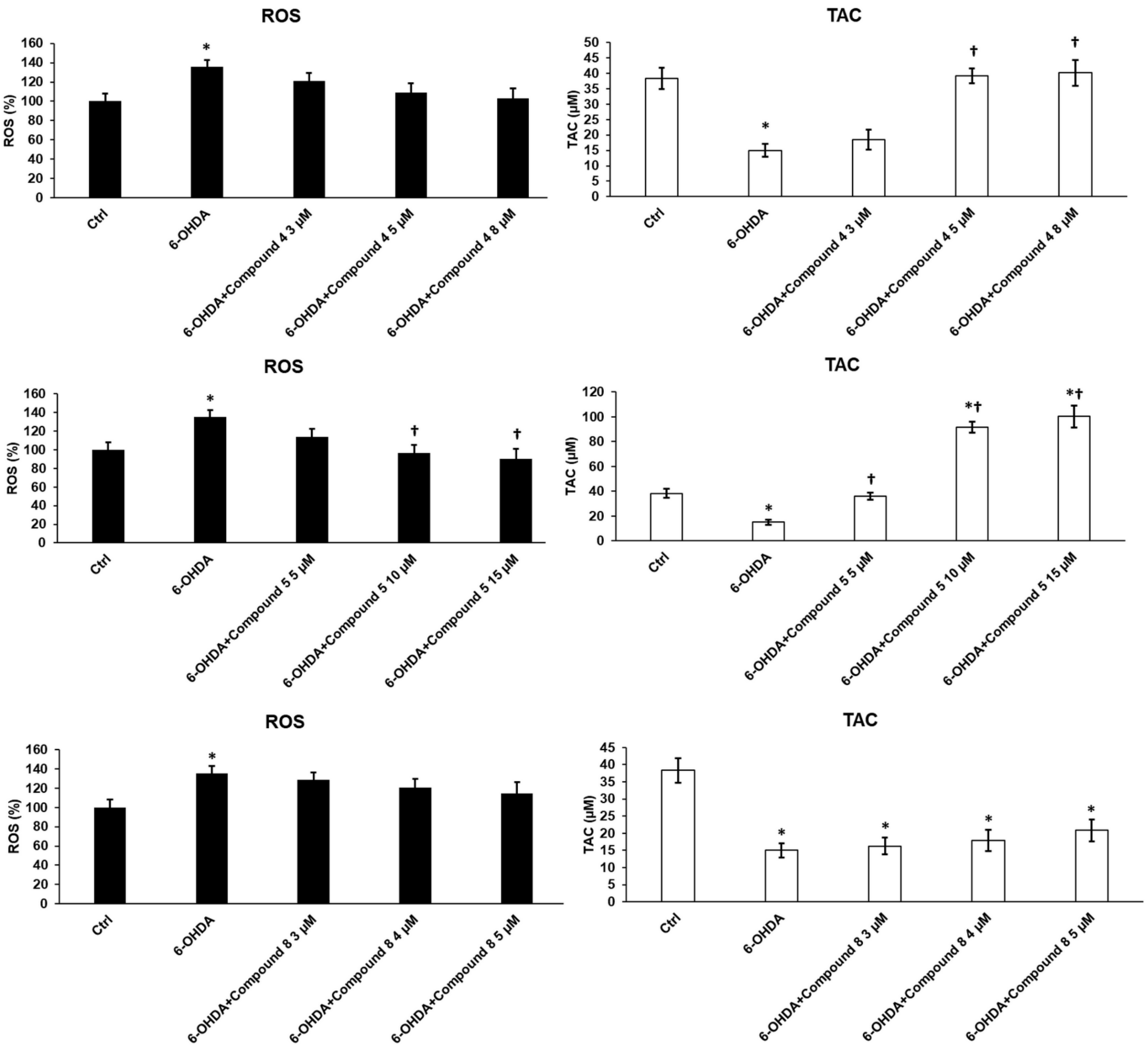
ROS
ROS (%)
160
140
120
100
80
60
40
20
0
*
Ctrl
6-OHDA
6-OHDA+Compound 4 3 µM
6-OHDA+Compound 4 5 µM
6-OHDA+Compound 4 8 µM
TAC
TAC (µM)
50
45
40
35
30
25
20
15
10
5
0
*
†
†
Ctrl
6-OHDA
6-OHDA+Compound 4 3 µM
6-OHDA+Compound 4 5 µM
6-OHDA+Compound 4 8 µM
ROS
ROS (%)
160
140
120
100
80
60
40
20
0
*
†
†
Ctrl
6-OHDA
6-OHDA+Compound 5 5 µM
6-OHDA+Compound 5 10 µM
6-OHDA+Compound 5 15 µM
TAC
TAC (µM)
120
100
80
60
40
20
0
*
†
*†
*†
Ctrl
6-OHDA
6-OHDA+Compound 5 5 µM
6-OHDA+Compound 5 10 µM
6-OHDA+Compound 5 15 µM
ROS
ROS (%)
160
140
120
100
80
60
40
20
0
*
Ctrl
6-OHDA
6-OHDA+Compound 8 3 µM
6-OHDA+Compound 8 4 µM
6-OHDA+Compound 8 5 µM
TAC
TAC (µM)
45
40
35
30
25
20
15
10
5
0
*
*
*
*
Ctrl
6-OHDA
6-OHDA+Compound 8 3 µM
6-OHDA+Compound 8 4 µM
6-OHDA+Compound 8 5 µM

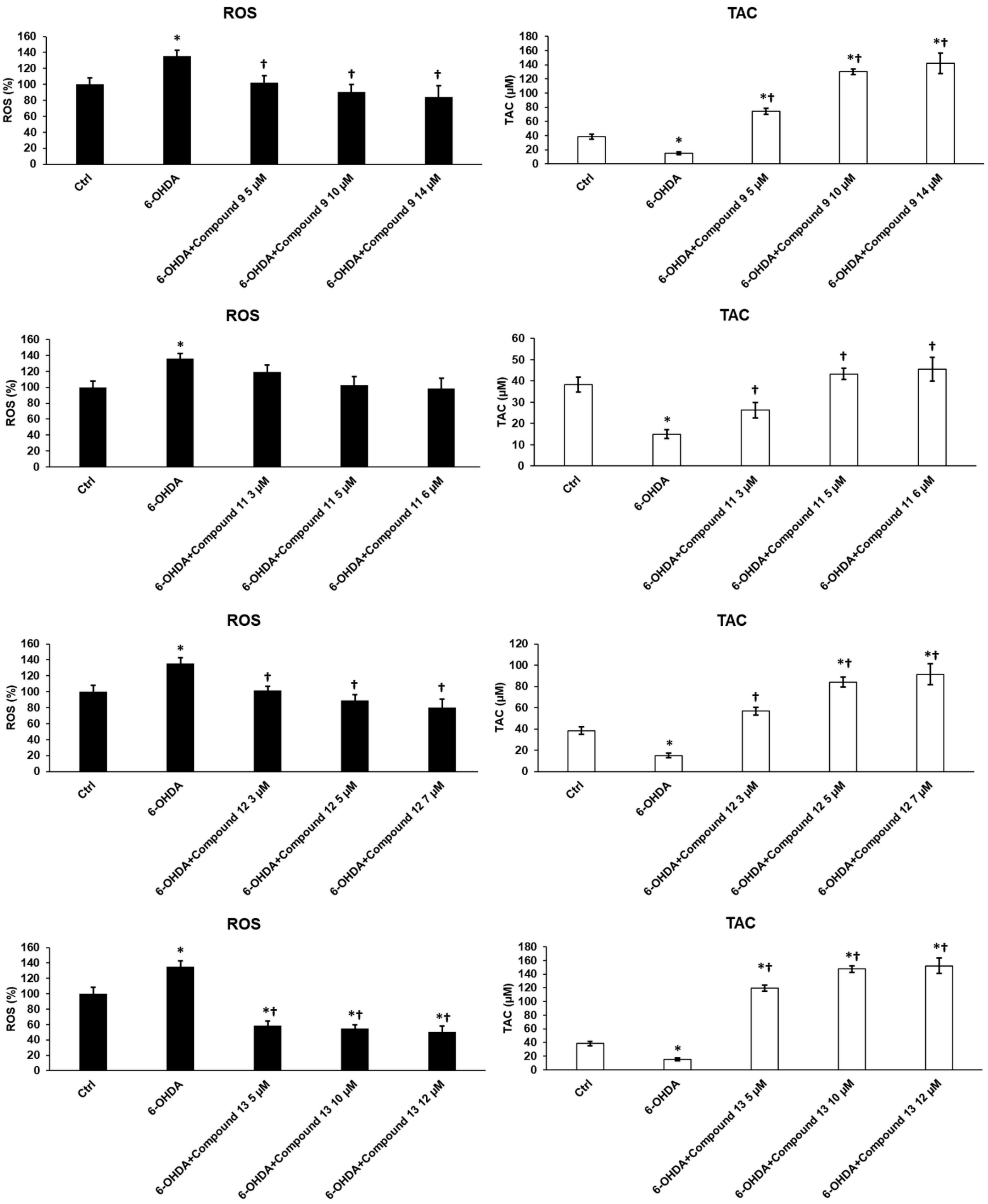

Supplementary Figure S3. Determination of ROS production and total antioxidant capacity in 6-OHDA-induced differentiated SH-SY5Y cells using three concentrations of test compounds. ROS determination was carried out using a Fluorometric Intracellular ROS Kit. ROS levels were compared to the control cells and expressed as a percentage. The TAC levels were determined using an Antioxidant Capacity Assay Kit. Each column shows the mean ± SD derived from three independent experiments (n = 3). The * signifies a p-value of less

than 0.05 in comparison to the control. The † indicates $p < 0.05$, compared with the 6-OHDA treatment. Abbreviations: 6-OHDA, 6-hydroxydopamine; ROS, reactive oxygen species.
