# Supplementary figures and images for: Synthetic Cyclic C5-Curcuminoids Increase Antioxidant Defense and Reduce Inflammation in 6-OHDA-Induced Retinoic Acid-Differentiated SH-SY5Y Cells
Source: Antioxidants (Basel). 2025 Aug 28;14(9):1057. doi: 10.3390/antiox14091057 (PMC12466566; doi:10.3390/antiox14091057)

## Compound 5:

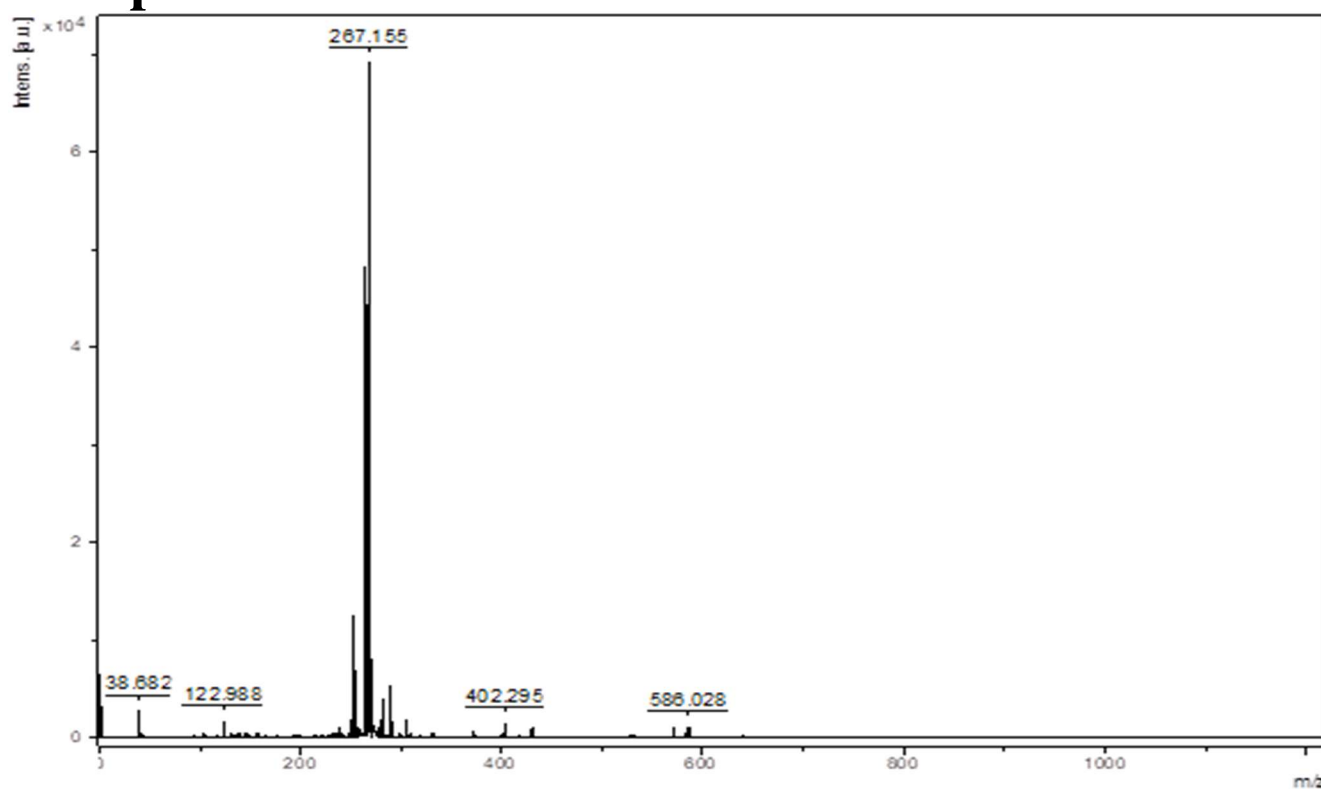

## Compound 13:

HI-185-1018 #551 RT: 5.43 AV: 1 NL: 5.74E8  
T: FTMS + c ESI Full lock.ms [110.0000-1000.0000]

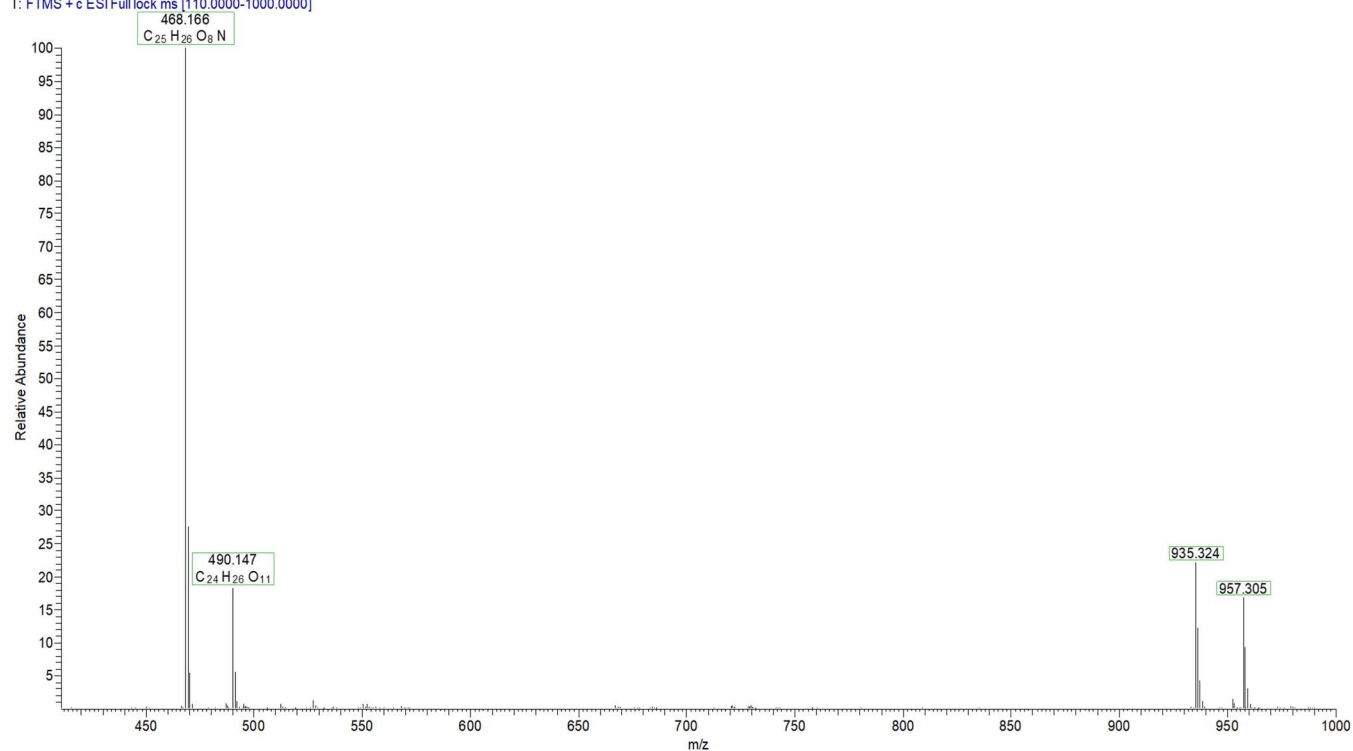

Supplement: Supplementary file 1 [file antioxidants-14-01057-s001.zip › Figure S1.pdf]

# Compound 5:

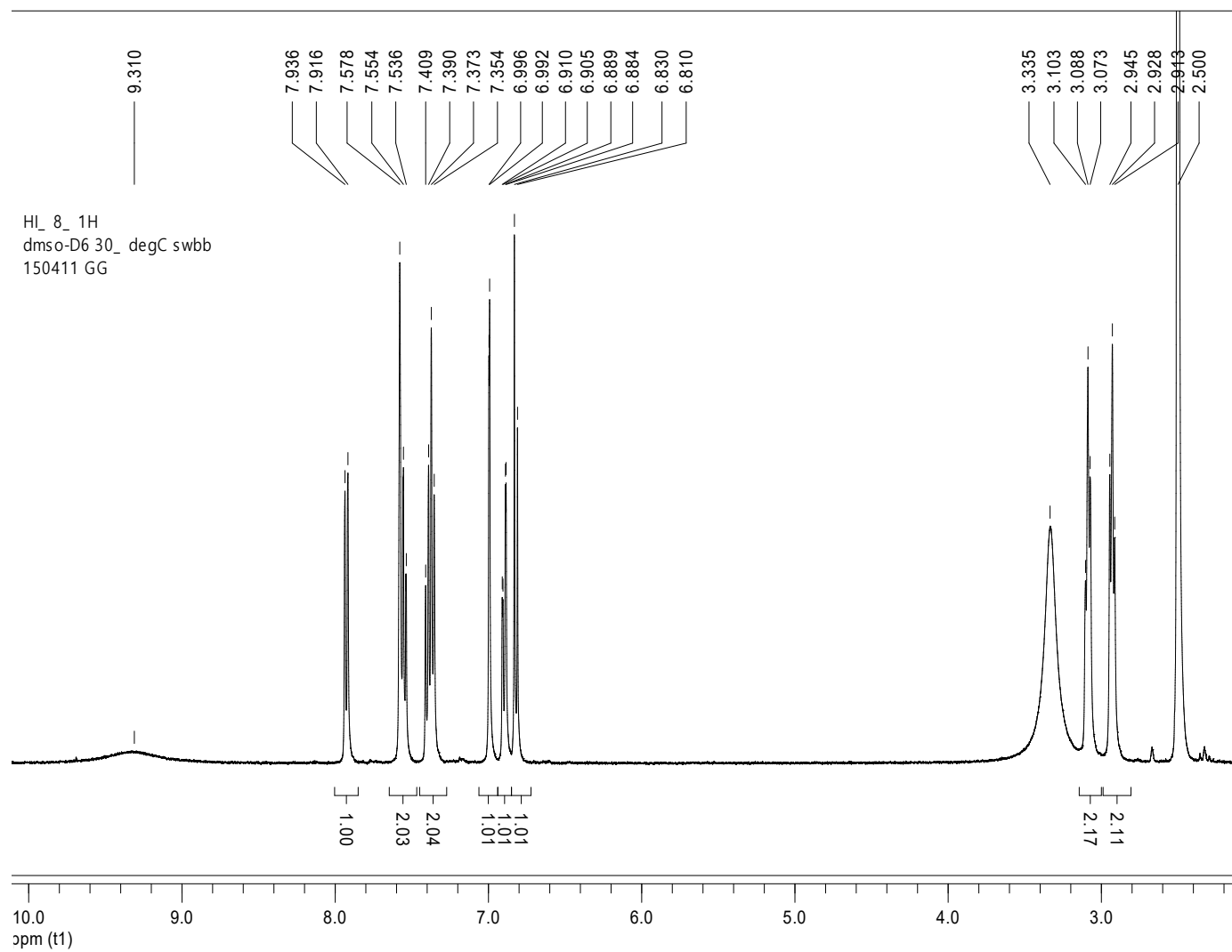

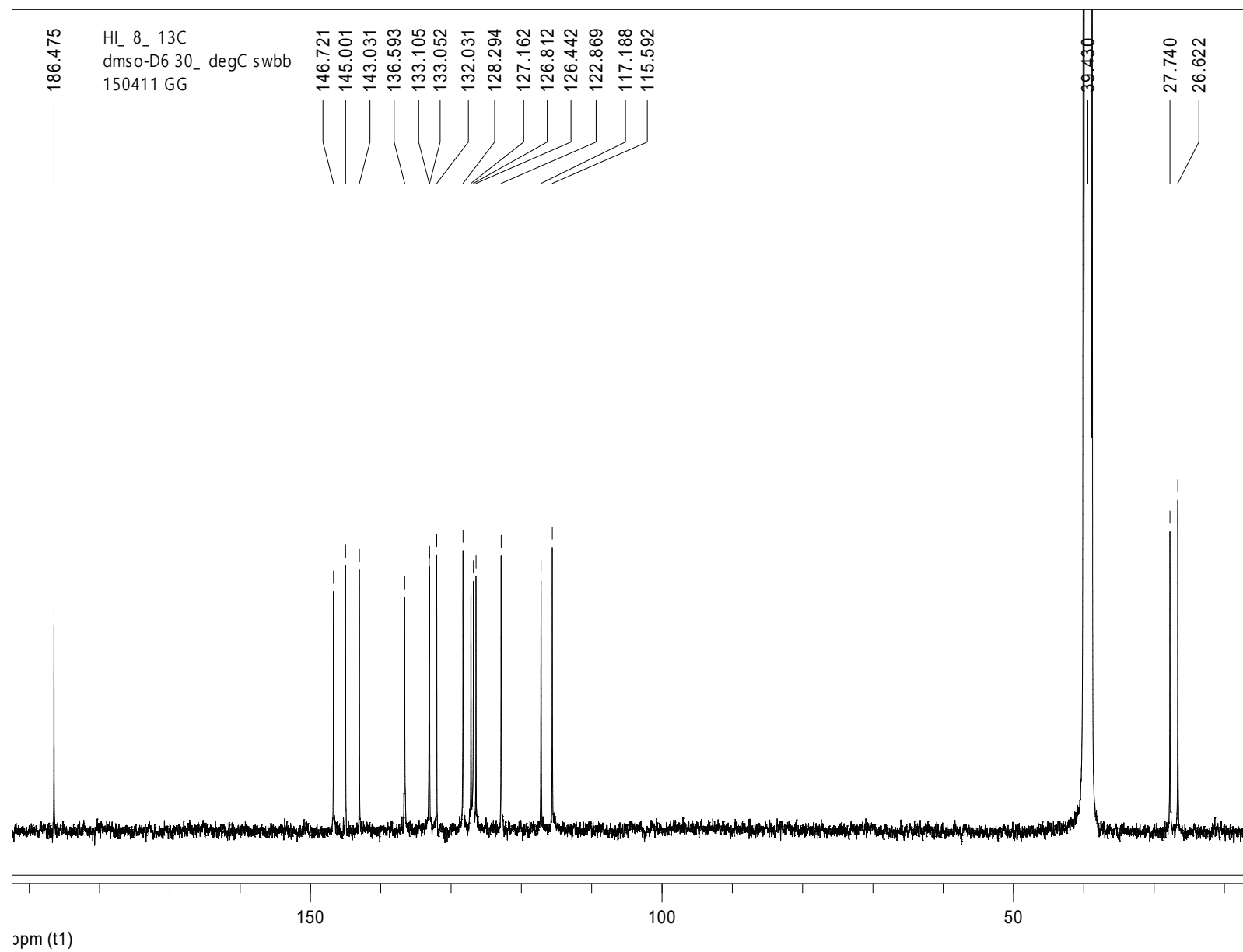

# Compound 13:

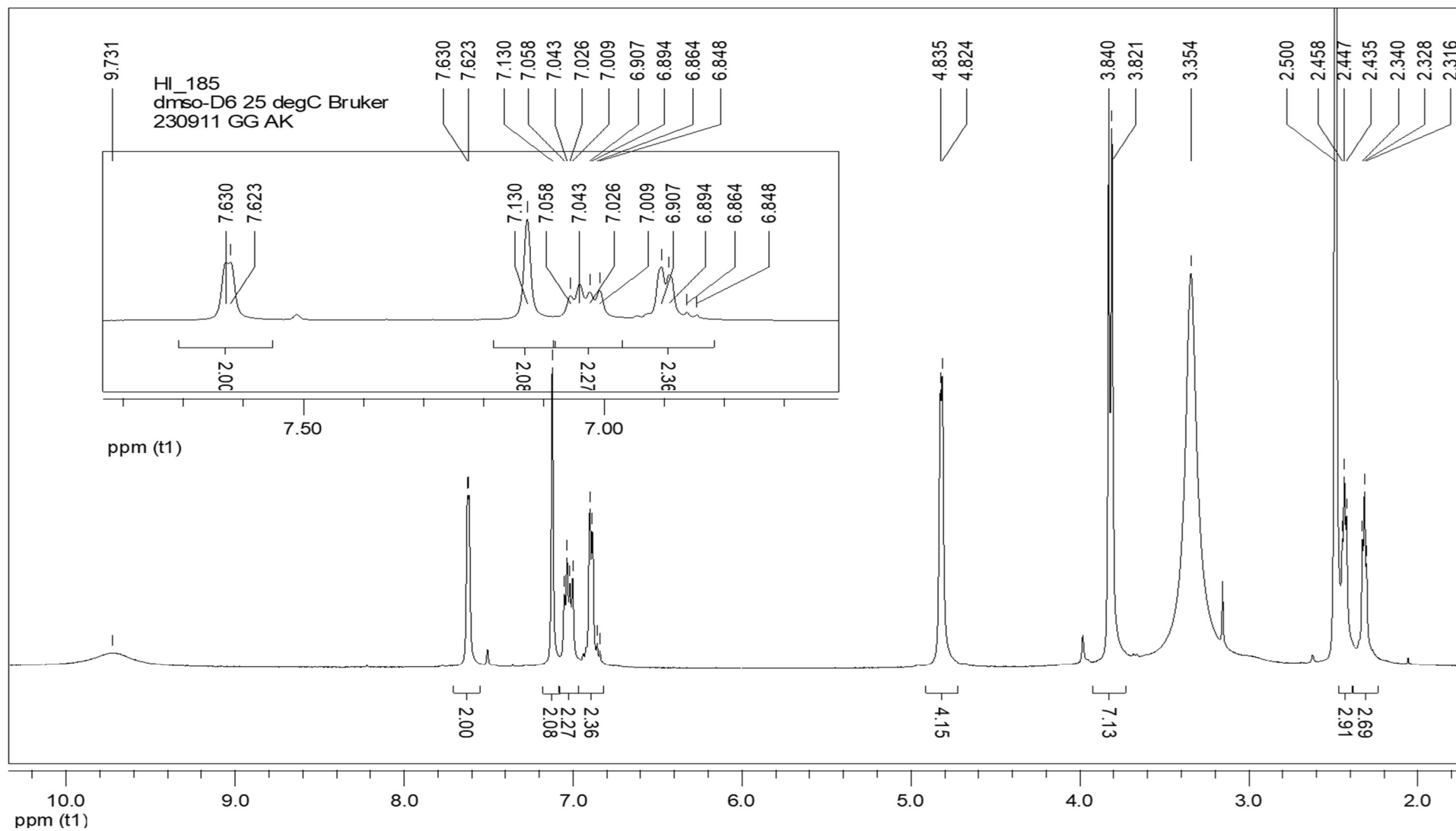

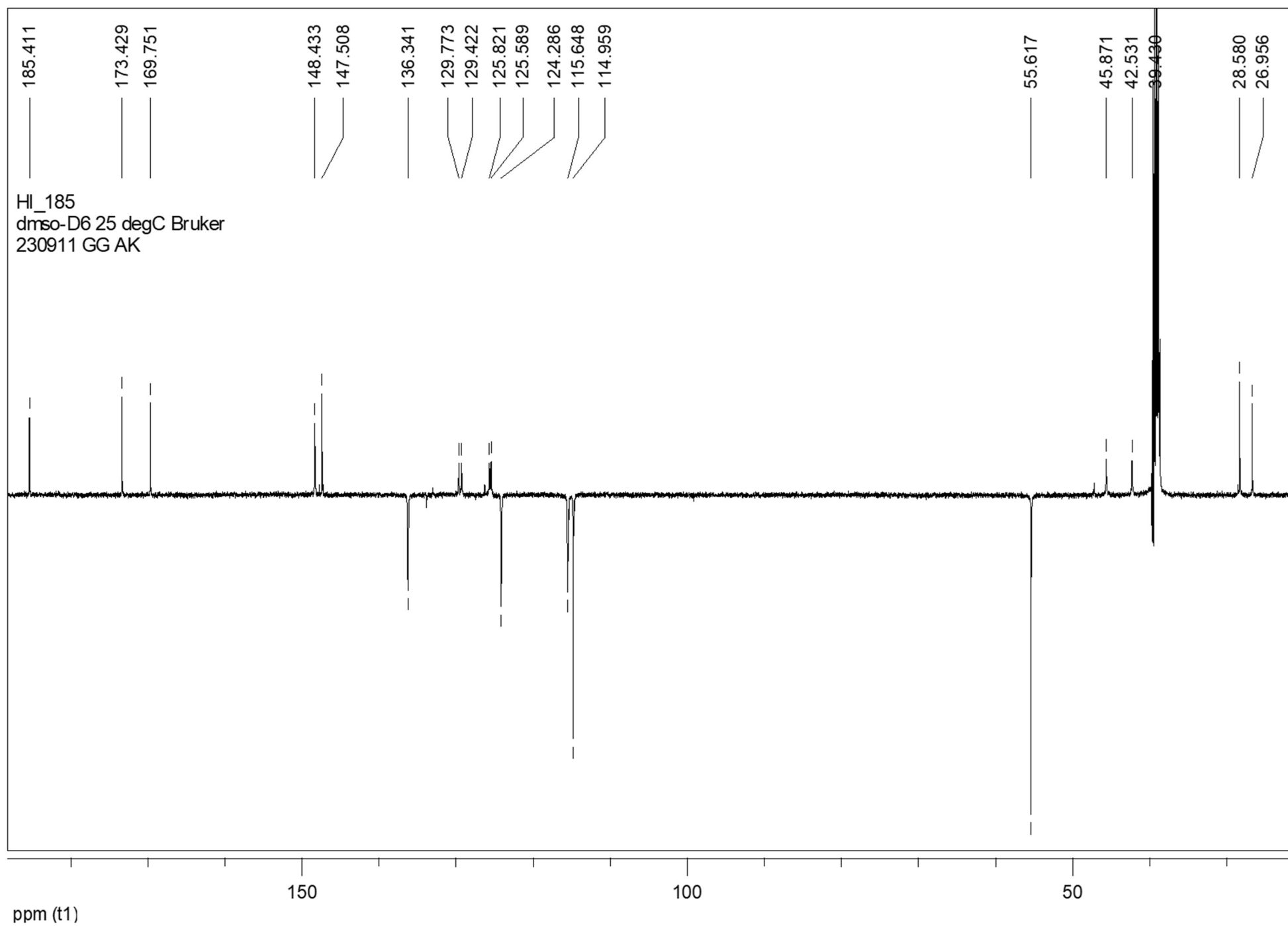

Supplement: Supplementary file 1 [file antioxidants-14-01057-s001.zip › Figure S2.pdf]
